# Pharmacological Effects of the Ruthenium Complex NAMI-A Given Orally to CBA Mice With MCa Mammary Carcinoma

**DOI:** 10.1155/MBD.2001.1

**Published:** 2001

**Authors:** Sonia Zorzet, Alenka Sorc, Claudia Casarsa, Moreno Cocchietto, Gianni Sava

**Affiliations:** 1 Department of Biomedical Sciences University of Trieste via L. Giorgieri 7 Trieste I-34127 Italy; 2 Fondazione Callerio Onlus via A. Fleming 22-31 Trieste I-34127 Italy

## Abstract

NAMI-A, imidazolium *trans*-imidazoledimethylsulfoxidetetrachlororuthenate, is a ruthenium based compounds capable of inhibiting the growth of lung metastases of solid tumours in a number of experimental conditions.The aim of this study was to investigate the potential use of NAMI-A by the oral route to treat lung metastases of MCa mammary carcinoma in the CBA mouse. treatment of mice, carrying intramuscular tumours in advanced stage of growth, for 11 consecutive days caused a significant reduction of the weight of lung metastases over the range of doses from 150 to 600 mg/kg/day. No sign of toxicity was observed at the histological analysis in the gut epithelium or in the kidney parenchyma, and NAMI-A concentration in the kidney was more than 10-fold lower than after intraperitoneal treatments. NAMI-A is thus active against metastases also by the oral route, suggesting the use of this way to treat tumour bearing hosts for long periods.


### PHARMACOLOGICAL EFFECTS OF THE RUTHENIUM COMPLEX NAMI-A GIVEN ORALLY TO CBA MICE WITH MCa MAMMARY CARCINOMA

Sonia Zorzet[1], Alenka Sorc[2], Claudia Casarsa[2], Moreno Cocchietto[2], and Gianni Sava*[1,2]

[1]Department of Biomedical Sciences, University of Trieste, via L. Giorgieri 7

[2]Fondazione Callerio Onlus, via A. Fleming 22-31, I-34127 Trieste, Italy.

**ABSTRACT**

NAMI-A, imidazolium *trans*-imidazoledimethylsulfoxidetetrachlororuthenate, is a ruthenium based compounds capable of inhibiting the growth of lung metastases of solid tumours in a number of experimental conditions. The aim of this study was to investigate the potential use of NAMI-A by the oral route to treat lung metastases of MCa mammary carcinoma in the CBA mouse. Treatment of mice, carrying intramuscular tumours in advanced stage of growth, for 11 consecutive days caused a significant reduction of the weight of lung metastases over the range of doses from 150 to 600 mg/kg/day. No sign of toxicity was observed at the histological analysis in the gut epithelium or in the kidney parenchyma, and NAMI-A concentration in the kidney was more than 10-fold lower than after intraperitoneal treatments. NAMI-A is thus active against metastases also by the oral route, suggesting the use of this way to treat tumour bearing hosts for long periods.

### INTRODUCTION

Metastases of solid tumours represent the most important target for therapy in that they are the most frequent responsible for the failure of cancer therapy. Metastases are always scattered, often in more than one vital organ, and are not erasable by conventional surgery and/or radiotherapy. On the other hand, cytotoxic cancer chemotherapy fails against solid tumour metastases in that, among all, metastases differ from the primary tumours of origin because of poor sensitivity to conventional drugs [1-3]. Drugs active against metastases are therefore strongly desired. These drugs should show the capacity of inhibiting metastasis growth (i.e. the growth of metastases already formed), besides metastasis formation (i.e. the steps that bring tumour cells to form distant metastases) [4]. It is thus important to get the so-called antimetastasis agents rather than the mostly known antimetastatic drugs, provided that in human beings the most probable drug treatment is for already established metastases. In recent years, we accumulated a strong evidence for the effects of some ruthenium complexes on solid tumour metastases in mice bearing experimental tumours [5-7]. Of these complexes, the one called NAMI-A (imidazolium *trans*-imidazoletedimethylsulfoxidetrachlororuthenate) showed adherence to the above characteristics: it is active against spontaneous metastases of solid tumours also when drug treatment occurs in mice with advanced tumours, i.e. at a time at which metastases are already settled in the lungs [5,8]. Moreover, NAMI-A appears to control metastasis growth by a mechanism unrelated to direct cell cytotoxicity, typical of conventional chemotherapy agents, still showing effects similar or even superior to those of a drug of wide clinical use such as cisplatin [7,9-11]. More recently, NAMI-A, though undergoing a series of molecular metabolisms in aqueous solutions, was shown capable of maintaining its antimetastatic activity in a broad range of experimental conditions, thus suggesting the possibility of handling its clinical management in a rather flexible way [12].

The aim of the present investigation was therefore that of examining the effects of NAMI-A given by the oral route to mice bearing the solid metastasising tumour MCa mammary carcinoma. The efficacy of NAMI-A by this route of administration should facilitate the treatment of tumour bearing hosts to cover a relatively long period of time, as probably required for the metastatic pathology. In fact, provided that NAMI-A has repeatedly shown efficacy against metastases with a mechanism involving the alteration of the cancer cells with host cells/tissues [13], the reduction of metastases till their complete removal might require a long exposure of the tumour cells to the drug. In this respect, the oral route may meet this requirement, particularly if a better compliance is also proved by the host. This study therefore examines the effects of NAMI-A on lung metastasis formation in the light of the effects of the complex on primary tumour, on gut epithelium and kidney and on ruthenium uptake by these tissues.

### MATERIALS AND METHODS

**Compounds and treatment**. NAMI-A was prepared according to a patented procedure (Mestroni et al., 1998). The drug was administered orally admixed throughout the powered food. Animal treatment was performed on days 10-21 after tumour implantation. All the animals were pre-conditioned to powdered food for 2-weeks before the experiment. Drug concentration was selected to provide 100, 150, 200, 300 and 600 mg/kg/die on the basis of an average daily food consumption of 5.0±0.1 g per mouse, determined in the pre-conditioning period. Food consumption remained constant throughout the duration of the experiment in either Controls or in the group receiving NAMI-A.

**Tumour line**. MCa mammary carcinoma cells (grown in CBA mice) were used for *in vivo* testing. CBA mice were obtained from a locally established breeding colony grown according to the standard procedures for inbred strains. The tumour graft procedure for MCa mammary carcinoma were the same as those already described in detail [14]. Briefly, tumour cells of a single cell suspension were prepared from mincing with scissors the primary tumour masses obtained from donors similarly implanted 2-weeks before. $10^6$ single viable tumour cells were injected i.m. into the left hind leg calves of mice in groups of 8-10 animals.

Animal studies were carried out according to the guidelines in force in Italy (DDL 116 of 21/2/1992) and in compliance to the Guide for the Care and Use of Laboratory Animals, Department of Health and Human Services Publication No. (NIH)86-23, Bethesda, MD, National Institutes of Health, 1985.

**Primary tumour growth and lung metastasis evaluation**. Primary tumour growth was determined by calliper measurements of two orthogonal axes and the tumour volume was calculated by the formula: $(\pi/6) \times a^2 \times b$, wherein **a** is the shorter and **b** is the longer axis; the tumour density was assumed to be equal to one. Lung metastases were counted by carefully examining the surface of the lungs, immediately after killing of the animals by cervical dislocation. Lungs were dissected into the five lobes, washed with PBS and examined under a low power microscope equipped with a calibrated grid. The weight of each metastasis was calculated by applying the same formula as for primary tumours and the sum of each individual weight gave the total weight of the metastatic tumour per animal.

**Histological analysis**. Sections for light microscopy were prepared from paraffin embedded kidneys and gut (from ileum) sections, which were removed, washed in water and fixed in 10% formaline, and processed according to the standard procedure for inclusion and following rehydration (xylene, alcohol, water), with sections cut at 6 μm. Sections were stained with Cajal-Gallego mounted in Canada Balsam and were observed with a Leitz-Orthoplan microscope. Examinations were made on three different slides, each containing three slices for each sample.

**AAS of ruthenium**. Ruthenium was measured in triplicate by atomic absorption spectroscopy using a Varian SpectrAA-300 instrumentation supplied with a graphite furnace mod GTA-96, an autosampler mod PSD-96, and a specific ruthenium emission lamp (Hollow cathod lamp Varian P/N 56-101447-00). Ruthenium was measured in samples of 10 μl at 349.9 nm with an atomising temperature of 2,500°C, using argon as purge gas at the flow rate of 3.0 l/min. Before daily analysis, a five point calibration curve was performed by Ruthenium Custom-Grade Standard 998 μg/ml in 3.3% HCl (Inorganic Ventures Inc., Lakewood, NJ, USA).

**Statistical analysis**. Experimental raw data were submitted to computer-assisted statistical analysis using ANOVA analysis of variance and Tukey-Kramer post-test.

### RESULTS AND DISCUSSION

The effects of NAMI-A, given orally to mice bearing MCa mammary carcinoma in advanced stage of growth, on lung metastasis formation are shown in Figure 1. On metastasis number, NAMI-A caused the reduction to about 55-60% of untreated controls at any dose tested from 150 to 600 mg/kg/day; the submission of these results to the one-way analysis of variance showed a significant reduction at the doses of 150 and 600 mg/kg/day, indicating that the rather wide variation observed in the treated groups reduced the statistical significance at the other doses.

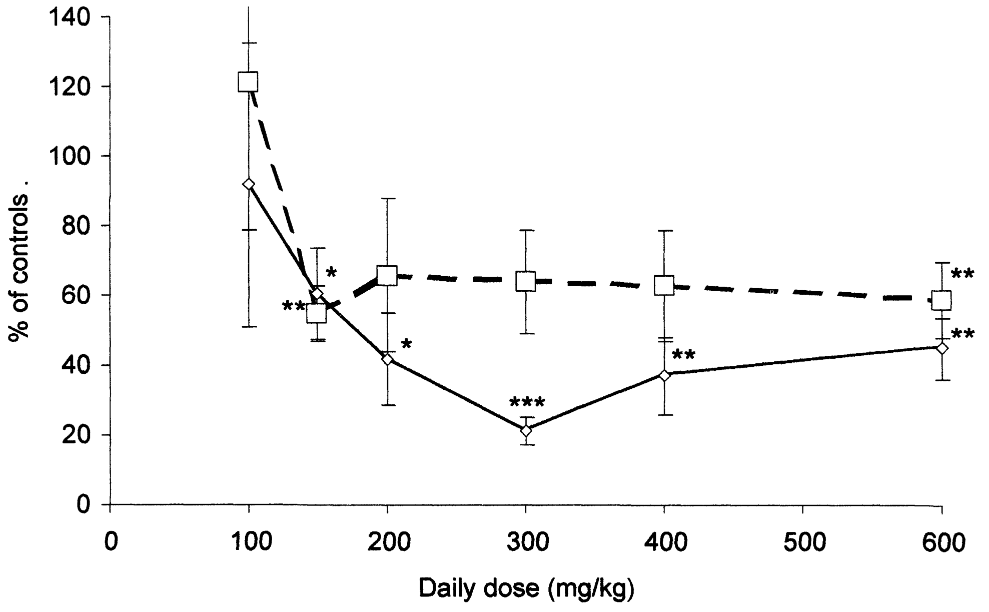

Figure 1. Effects of the oral treatment with NAMI-A on the growth of lung metastasis.

Groups of 8 CBA female mice, implanted i.m. with $10^6$ MCa mammary carcinoma cells on day 0, were given daily NAMI-A, at the above daily doses, orally on days 10-21. Mice were killed and lung metastases counted on day 23. Each value is the mean±S.E. obtained in each group (---: metastasis number, —: metastasis weight). Data were obtained in four separate experiments, each performed with appropriate untreated controls fed with the same type of food without NAMI-A; in detail, the results of the doses of 100, 200, 300 and 400 mg/kg were repeated twice in two repeated experiments. The representative values obtained in control mice are: 28.4±3.0 – 37.4±7.2 (metastasis number), and 97.2±17.6 - 128±22.3 (metastasis weight). *$p<0.05$, **$p<0.01$, ***$p<0.001$ vs the controls of the respective experiments.

Conversely, on metastasis weight NAMI-A resulted active with any dose used from 150 mg/kg/day onward (Figure 1), and the reduction was around 40% of untreated controls. For comparison, the i.p. treatment at different doses and schedules showed a similar trend (i.e. marked inhibition of metastasis weight). However, the extent of metastasis reduction was generally superior being around 10-20% of untreated controls [5,8].

The effects on primary tumour growth (similarly to the i.p. treatment and independently of the dose-level used) was much less evident and statistically not significant (data not reported). Similarly, none of the doses used for the oral treatment caused host toxicity, as determined by the variation of body weight gain between the start and the end of treatment in comparison to untreated controls.

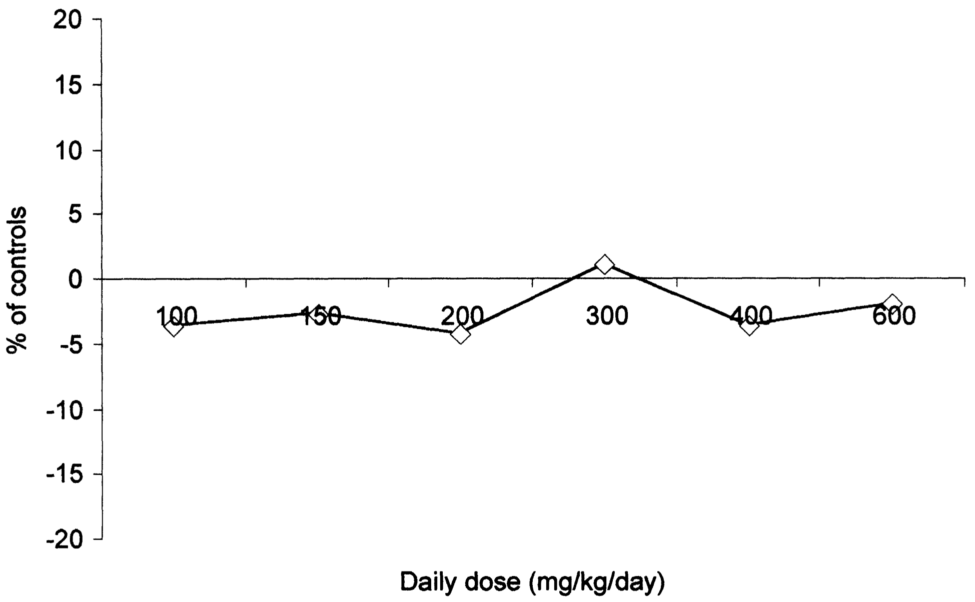

Figure 2. Effects of the oral treatment with NAMI-A on the body weight of animals.

Body weight variation between the beginning and the end of treatment in the groups of mice receiving NAMI-A was expressed as % versus untreated controls. None of the report

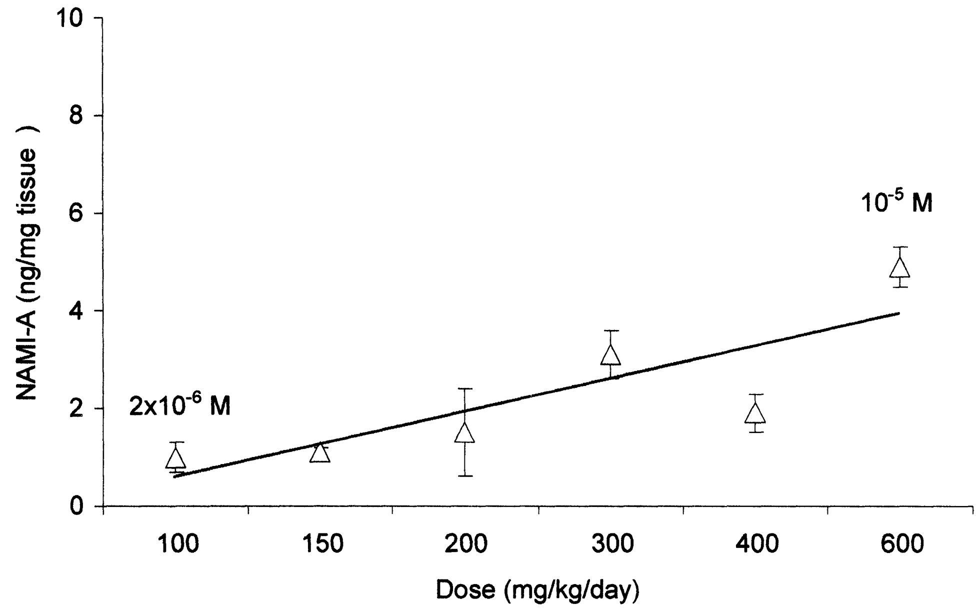

Figure 3. Ruthenium concentration in the kidney of mice of Figure 1.
ed differences can be considered statistically significant.

The measurement of ruthenium in the kidney of mice treated orally with NAMI-A shows that the concentration of the metal (referred as NAMI-A) increases proportionally to the daily dose used (Figure 3). Indeed, the amount of NAMI-A found in the kidney of these animals was markedly lower than after i.p. administration. By comparison we may report the data obtained with the dose of 35 mg/kg/day given daily for 6 consecutive days. In this case, the amount of NAMI-A found in the kidney was approximately 10 to 20-fold greater than that reported in Figure 3, at the higher dose of 600 mg/kg/day for 11 consecutive days.

The kidneys of mice of Figure 1 were processed for flameless atomic absorption spectroscopy analysis of ruthenium content. Each value is the mean±S.E. obtained from at least three separate samples obtained from three different mice per group.

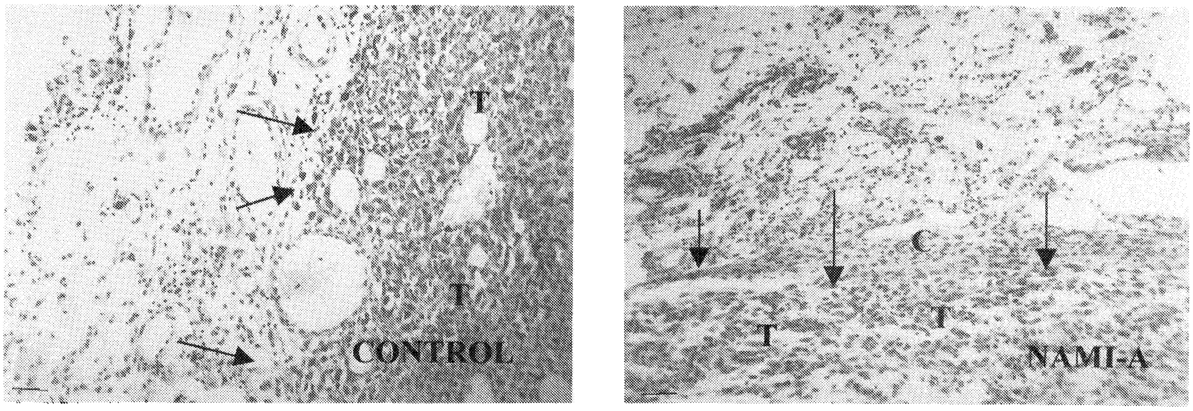

Figure 4. Histological appearance of tumour parenchyma of mice treated with 200 mg/kg/day NAMI-A for 11 consecutive days.
C: connective tissue; T: tumour mass; arrows show the external border of the tumour mass.

The oral treatment with NAMI-A induces a marked modification of the appearance of tumour parenchyma, particularly at the external border which appears more regular at the interface with the surrounding tissue and with an increased density of connective tissue. Apparently, tumour cells do not appear to be modified by the treatment with NAMI-A and their density is similar to that of the control samples. At a greater magnification it is possible to appreciate better the changes at the border of the primary tumour mass on which several inflammatory cells are distinguishable. These alteration are perfectly similar to those observed following i.p. treatment of this tumour [5]. A detailed examination of the cells of Figure 5 shows the presence of infiltrating leukocytes, particularly PMNs and lymphocytes (shown by arrows), already evidenced following the i.p. treatment in primary tumour masses of both i.m. and s.c. growing tumours [13].

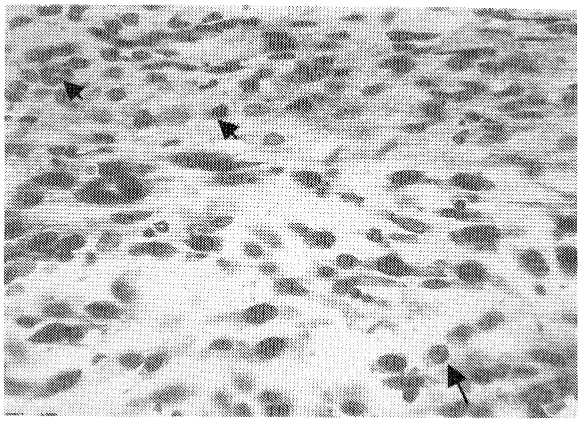

Figure 5. Histological appearance of tumour parenchyma of mice treated with NAMI-A. Higher magnification (sector **C**) of Figure 4. Collagen fibres are coloured in grey-blue and appear scattered among the tumour cells in the third top of the figure. Arrows show some infiltrating leukocytes (Top left: PMN; middle: lymphocyte; right down: monocyte)

The examination of gut epithelium and kidney parenchyma, of mice treated orally with NAMI-A, by histological staining and light microscopy analysis, showed no appreciable alteration of the architecture of the tissues examined (Figure 6). Gut epithelium was regular and the only change consisted of an increased density of connective tissue on the basal membrane over untreated controls. Kidney tubules and glomeruli appear regular with cells well defined and no presence of cell suffering, dilation of tubules or swelling of the extracellular matrix.

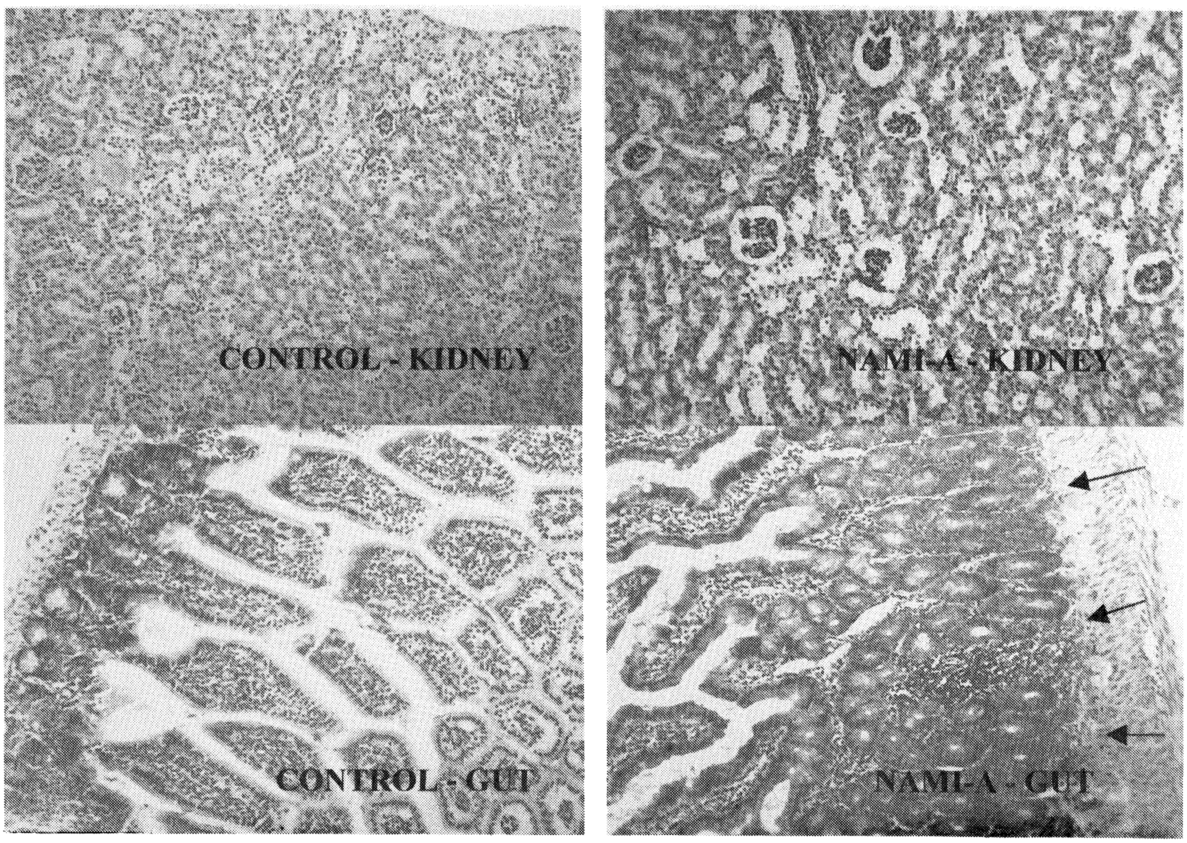

Figure 6. Histological appearance of kidney parenchyma (top) and gut epithelium (bottom) of control mice (left) and of mice treated with NAMI-A (right). Slices were prepared from mice of the experiments of Figure 1 and represent a picture present independently of the dose of NAMI-A used and of the experiment chosen. The evaluation of the histological changes induced by NAMI-A was made in a single blind experiment with at least three different glasses, each mounted with three independent slices, per animal. Arrows highlight the increased thickness of collagen fibres under basal membrane.

CONCLUSION

Among the most important promising ruthenium complexes appeared in the literature [15-16], those with sulfoxide ligands still continue to show the better pharmacological properties against solid tumour metastases.

NAMI-A proved to be active against solid tumour metastases also when given by the oral route over a 10 day period to mice with advanced MCa mammary carcinoma. Although the overall effect is slightly less pronounced than that obtained following i.p. treatment, it must be stressed that by this route of administration the kidney concentration of the compound is markedly lower and no histological toxicity is evidenced. The lack of toxicity at the gut level and on kidneys suggest the possibility to use NAMI-A by this route also for periods longer than that used in the present study. Provided that to keep metasases under full control by non cytotoxic drugs such as NAMI-A, they should be treated for long periods, these data suggest the feasibility of this treatment and its effectiveness.

**Acknowledgements:** Work supported by Laboratorio per Investigare Nuovi Farmaci Antimetastasi (LINFA), and done in the framework of the project "Pharmacological and Diagnostic Properties of Metal Complexes" and the contribution of the EU COST D8/97/0017 Project. The technical assistance of M. Zabucchi is gratefully appreciated.

REFERENCES

1. Aslakson CJ and Miller FR. *Cancer Res* **52**: 1399-1405, 1992.
2. Leibovici J, Klorin G, Huszar M, Hoenig S and Michowitz M. *Int J Exp Path* **72**: 139-150, 1991.
3. Liotta LA and Stetler-Stevenson WG. In: *Cancer: Principles and Practice of Oncology* (DeVita VT, Hellman S and Rosenberg SA, eds.), JB Lippencott Co., Philadelphia, 134-149, 1993.
4. Sava G and Bergamo A. *Anticancer Res* **19**: 1117-1124, 1999.
5. Sava G, Capozzi I, Clerici K, Gagliardi R, Alessio E and Mestroni G. *Clin Exp Metastasis* **16**: 371-379, 1998.
6. Bergamo A, Cocchietto M, Capozzi I, Mestroni G, Alessio E and Sava G. *Anticancer Drugs* **7**: 697-702, 1996.
7. Bergamo A, Gagliardi R, Scarcia V, Furlani A, Alessio E, Mestroni G and Sava G. *J Pharmacol Exp Ther* **289**: 559-564, 1999.
8. Sava G, Clerici K, Capozzi I, Cocchietto M, Gagliardi R, Alessio E, Mestroni G and Perbellini A. *Anticancer Drugs* **10**: 129-138, 1999.
9. Sava G, Pacor S, Bergamo A, Cocchietto M, Mestroni G and Alessio E. *Chem-Biol Interact* **95**: 109-126, 1995.
10. Capozzi I, Clerici k, Cocchietto M, Salerno G, Bergamo A and Sava G. *Chem-Biol Interact* **113**: 51-64, 1998.
11. Zorzet S, Bergamo A, Cocchietto M, Sorc A, Gava B, Alessio E, Iengo E and Sava G. *J Pharmacol Exp Ther* **295**: 927-933, 2000.
12. Sava G, Bergamo A, Zorzet S, Gava B, Casarsa C, Cocchietto M, Furlani A, Scarcia V, Serli B, Iengo E, Alessio E and Mestroni G. *JBCI* (submitted manuscript).
13. . Magnarin M, Bergamo A, Carotenuto ME, Zorzet S and Sava G. *Anticancer Res* **20**: 2939-2944, 2000.
14. Poliak-Blazi M, Boranic M, Marzan B, Radacic M. *Vet Arh* **51**: 99-107, 1981.
15. Clarke MJ. In: *Metal Ions in Biological Systems* (Sigel H ed.), Marcel Dekker, New York, 231-383, 1989.
16. Keppler BK, Rupp W, Juhl UM, Endres H, Niebl R and Balzer W. *Inorg Chem* **26**: 4366-4370, 1987.

